# Factors affecting the development of complications in Crohn’s disease in patients undergoing intestinal resection

**DOI:** 10.1097/MD.0000000000032957

**Published:** 2023-02-22

**Authors:** Nazim Gures, Server Sezgin Uludag, Ergin Erginoz, Suleyman Yildirim, Yusuf Ziya Erzin, Kagan Zengin

**Affiliations:** a Department of General Surgery, Balikesir Ataturk City Hospital, Balikesir, Turkey; b Department of General Surgery, Istanbul University Cerrahpasa – Cerrahpasa School of Medicine, Istanbul, Turkey; c Department of Internal Medicine, Istanbul University Cerrahpasa – Cerrahpasa School of Medicine, Istanbul, Turkey.

**Keywords:** complications, Crohn disease, intra-abdominal disease, surgery

## Abstract

Surgery is a common form of management for Crohn disease (CD) in the presence of intra-abdominal complications. In this study, we investigated the effect of various factors on the development of postoperative complications in patients who underwent surgery for complicated CD. Patients who underwent surgery between 2011 and 2018 were included in this study. Information on age, sex, presence of extraintestinal findings, operation indications, operation type, and postoperative complications was obtained. Groups with and without postoperative complications were compared according to body mass index, American Society of Anesthesiologists score, comorbidities, smoking status, preoperative drug use, presence of perianal disease, presence of a stoma, synchronous small intestine resection surgery, duration of hospital stay, and preoperative biochemical parameters. A total of 192 patients were included, of which 53.1% were female and 46.9% were male. Patients were indicated for surgery for reasons such as stricture, abscess, fistula, and tumor development. As the surgical method, patients were operated on by open or laparoscopic method (86% and 14%, respectively). Postoperative complications occurred in 30 female and 33 male patients (15.6% and 17.1%, respectively). Patient age, smoking status, steroid use, perianal disease, presence of stoma, and presence of extra intestinal findings were significantly higher in the complicated group. Surgery may be inevitable for CD in the presence of complications. In cases of patient age, smoking, steroid use, perianal disease, stoma opening, and presence of extra intestinal findings, patients with CD who undergo surgery should be followed up closely in terms of the development of complications.

## 1. Introduction

Crohn disease (CD) is a chronic disease characterized by transmural inflammation that can affect the entire gastrointestinal tract anywhere from the mouth to the anus, its incidence is increasing worldwide. It is accepted that the disease is formed by a complex process in which genetic predisposition, environmental factors, and altered intestinal flora cause changes in the innate and adaptive immune systems. In North America, its annual incidence is reported as 3.1 to 20.2 per 100,000 and its prevalence as 201 per 100,000.^[1–3]^ The most common symptoms include diarrhea, abdominal pain, rectal bleeding, fever, weight loss, and malaise. In addition, there may be extraintestinal findings such as aphthous stomatitis, uveitis, erythema nodosum, and inflammatory arthropathies.^[4]^

Therapies used in induction and remission maintenance are steroids, 5-aminosalicylic acid derivatives, immunomodulators, tumor necrosis factor (TNF) inhibitors, and new biological agents such as ustekinumab and vedolizumab, which have been used in recent years. Despite these treatments, approximately 2-thirds of the patients require lifelong surgical intervention. The most common causes of surgical treatment are intra-abdominal abscess, stricture and obstruction, perforation, bleeding, and cancer development.^[3,5]^

Various different factors are available in the development of postoperative complications in patients undergoing surgery for complicated CD. As an example, immunosuppressive agents such as anti-TNF-α, preoperative anemia, preoperative hypoalbuminemia, high C-reactive protein (CRP) values, family history, duration of disease, extent of the removed specimen, nutrition status, and presence of penetration are among the risk factors that may increase the risk of postoperative abdominal complications in CD.^[6–8]^ A thorough patient history taking is therefore essential in order to monitor patients that are at high risk of developing postoperative complications.

In this series, we aimed to share the results of patients in a tertiary center who were operated on for complications due to CD. In particular, we questioned the factors of patients with postoperative complications that caused the development of complications.

## 2. Methods

In the study, 192 patients who were operated on for CD in a tertiary center between February 2011 and December 2018 were included. This study was approved by the Istanbul University Cerrahpasa – Cerrahpasa School of Medicine Institutional Review Board (16161519-604.01.01-137560). The demographic data of all patients, the reasons for the operation, operation method and type (open vs laparoscopic), and postoperative complications were evaluated. The patients were divided into 2 groups as those with complications in the early and/or late periods (<30 days, > 30 days, respectively) (Group 1) and those without complications (Group 2). Group 1 had complications such as an anastomotic leak, ileus, intra-abdominal abscess, bleeding, lung infection, wound site infections and stomatal detachments that prolonged hospital stay, and incisional-parastomal hernias. Patients under 18 years of age were not included in the study. Chronic diseases requiring medication use (such as diabetes mellitus, asthma, and congestive heart failure) were included.

Group 1 and 2 cases were evaluated in terms of body mass index (BMI), American Anesthesia Association physical condition scoring American Society of Anesthesiologists (ASA) score, comorbidity, smoking, medical therapy 5-aminosalicylic acid, methotrexate, azathioprine (AZA), steroid, anti-TNF alpha use, the presence of perianal disease, the presence of extra intestinal findings, the time from the first diagnosis to the operation, whether concurrent small bowel resection was performed, the presence of stoma, the early and late development of the complication, the duration of hospitalization, laboratory parameters such as white blood cell count, neutrophil count, CRP, erythrocyte sedimentation rate, albumin, and LDH.

Patients who used antibiotics together with steroid therapy for 8 weeks prior to surgery, those who received sulfasalazine, AZA, and methotrexate within 6 to 8 weeks prior to surgery, those who received oral sulfasalazine within 2 weeks prior to surgery, and those who received anti-TNF alpha treatment 8 weeks prior to surgery were among the participants that took part in the study. Surgical decisions were made in a multidisciplinary meeting with the joint decision of different disciplines. All surgical interventions were performed by surgeons experienced in colorectal surgery. Surgical procedures were performed with an open or laparoscopic approach. After ileocecal resection, reconstruction was performed manually or with the help of a stapler as side-to-side iso-peristaltic anastomosis. According to the preoperative (radiological and clinical) and preoperative findings, ileocecal resection, right/ left hemicolectomy, small bowel resection, and subtotal colectomy procedures were performed. Intestinal resections included single-segment or multiple-segment resections. The opening of the ostomy was created according to the surgeon preference according to the operative findings (presence of an intraabdominal abscess, multiple fistulae), and the medical and general performance of the patients.

### 2.1. Statistical analysis.

In the descriptive statistics of the data, mean, standard deviation, median lowest, highest, frequency and ratio values were used. The distribution of variables was measured with the Kolmogorov–Smirnov test. Mann–Whitney test was used to analyze quantitative independent data. The Chi-square test was used in the analysis of qualitative independent data, and the Fischer test was used when the conditions of the Chi-square test were not met. The effect level was investigated by univariate and multivariate logistic regression. SPSS 26.0 software package (IBM Corp., Armonk, NY) was used for statistical analyses.

## 3. Results

In the study, 103 of 192 patients were female (53.1%), and 90 were male (46.9%). The average age was 40.6 ± 10.8 (range 20–67). The mean age at diagnosis of the patients was 29.4 ± 10.2 years. Data on demographic data, BMI, ASA scores, and accompanying factors of the patients are shown in Table 1.

**Table 1:** Demographic data of patients, BMI, ASA scores, and accompanying factors.

		Min–Max	Mean ± standard deviation/n-%
Age at diagnosis		14.0–60.0	29.4 ± 10.2
Age at operation		19.0–63.0	34.5 ± 9.9
Sex	Woman		102–53.1%
	Man		90–46.9%
BMI		15.8–32.9	23.7 ± 4.5
ASA	I		159%–82.8%
	II		24%–12.5%
	III		9%–4.7%
Comorbidity			39%–20.3%
Smoking status			93%–48.4%
Antibiotic use			36%–18.8%
5-ASA			81%–42.2%
MTX			9%–4.7%
AZA			105%–54.7%
Steroid			36%–18.8%
Anti TNF Alfa			75%–39.1%
Abscess during operation			57%–29.7%
Perianal disease			54%–28.1%
Stoma			75%–39.1%
Synchronous small bowel resection			27%–14.1%
Early complication			60%–31.3%
Late complication			18%–9.4%
Extraintestinal findings			48%–25.0%
WBC		4.3–12.1	7.5 ± 1.9
Neutrophil		2.5–8.9	5.4 ± 1.4
CRP		0.4–87.0	15.4 ± 19.1
ESR after 1 h		2.0–97.0	15.9 ± 16.3
Albumin		2.3–5.2	4.1 ± 0.6
LDH		110.0–278.0	189.7 ± 35.7
Time until operation (yr)		0.5–19.0	5.1 ± 4.6
Hospital stay duration		4.0–18.0	7.6 ± 3.1

ASA = American Society of Anesthesiologists, AZA = azathioprine, BMI = body mass index, CRP = C-reactive protein, ESR = erythrocyte sedimentation rate, MTX = methotrexate, TNF = tumor necrosis factor, WBC = white blood cell.

Eighteen cases operated for small bowel perforation were diagnosed by postoperative histopathological evaluation. Of those that required an operation, 90 patients had stricture, 15 patients had stricture and fistula, 18 patients had a treatment-resistant abscess, 21 patients had fistula and abscess, 18 patients had perforation, 12 patients had intra-abdominal mass and/or cancer, 12 patients were unresponsive to medical treatment, and 6 patients had multiple enteroenteric and enterocolic fistula. Of the patients, 165 were operated on by open approach and 27 were operated on laparoscopically. In addition to ileocecal resection in 153 patients, right hemicolectomy was performed in 15 patients, subtotal colectomy was performed in 9 patients and ileocecal resection was performed in 15 patients. As seen from the data, the ileocecal resection was the most common type of operation performed. Partial colon and/or small intestine resections were also performed. Operation indications, type of operation, and postoperative complications of the patients are shown in Table 2.

**Table 2:** Operation indications of the patients, type of operation, postoperative complications.

		Total % (n)
Surgery indication	Stricture	46.9 (90)
	Perianal fistula + abscess	10.9 (21)
	Treatment-resistant abscess	9.4 (18)
	Perforation	9.4 (18)
	Stricture. + perianal fistula	7.8 (15)
	Abdominal mass + cancer	6.3 (12)
	Unresponsive to medical treatment	6.3 (12)
	Enteroenteric fistula	3.1 (6)
Early complications	Surgical site infection	12.5 (24)
	Anastomotic leakage	6.3 (12)
	Intraabdominal abscess	4.7 (9)
	Ileus	4.7 (9)
	Lung infection	3.1 (6)
	Stoma retraction	1.6 (3)
Late complications	Ileus	3.1 (6)
	Incisional hernia	3.1 (6)
	Parastomal hernia	3.1 (6)
Type of operation	Open	86 (165)
	Laparoscopic	14 (27)
	Ileocecal resection	79.7 (153)
	Right hemicolectomy	7.8 (15)
	Ileocecal resection + segmental colon or small bowel resection	7.8 (15)
	Subtotal colectomy	4.7 (9)

Among the groups, smoking, antibiotic use, steroid use, presence of perianal disease, stoma opening during surgery, presence of extra intestinal findings, and high CRP were significantly higher in the group with complications (*P* < .05). The cutoff value for CRP in the patient group was 8.9 (sensitivity 81% and specificity 75%). The area under the curve value was 0.782 (*P* = .003). No statistically significant difference was found in both groups in terms of BMI, ASA score, additional disease, smoking, 5-aminosalicylic acid, methotrexate, AZA, steroid use, anti-TNF alpha use, whether concurrent small bowel resection was performed, early and late development of a complication, white blood cell count, neutrophil count, erythrocyte sedimentation rate, albumin and LDH values, time until the operation, and hospital stay (Table 3).

**Table 3:** Comparison of demographic, operative data and laboratory results of cases with and without complications.

		Complication (−)				Complication (+)			*P*
		Median + SD/n-%	Median		Min–Max	Median + SD/n-%	Median	Min–Max	
Age at diagnosis		28.3 ± 10.4	26.0		14.0–60.0	31.4 ± 9.7	30.0	18.0–58.0	.129^m^
Age at operation		33.3 ± 10.1	31.0		19.0–63.0	36.8 ± 9.3	36.0	23.0–59.0	.124^m^
Sex	Woman	72%–55.8%				30%–47.6%			.537^X²^
	Man	57%–44.2%				33%–52.4%			
BMI		24.0 ± 4.5	23.2		17.4–32.9	23.2 ± 4.6	21.8	15.8–32.9	.479^m^
ASA	I	111%–86.0%				48%–76.2%			.529^X²^
	II	12%–9.3%				12%–19.0%			
	III	6%–4.7%				3%–4.8%			
Comorbidity		18%–14.0%				21%–33.3%			.070^X²^
Smoking status		48%–37.2%				45%–71.4%			***.010*** ^X²^
Antibiotic use		12%–9.3%				24%–38.1%			***.006*** ^X²^
5-ASA		57%–44.2%				24%–38.1%			.643^X²^
MTX		9%–7.0%				0%–0.0%			.545^X²^
AZA		72%–55.8%				33%–52.4%			.796^X²^
Steroid		12%–9.3%				24%–38.1%			***.006*** ^X²^
Anti TNF Alfa		51%–39.5%				24%–38.1%			.912^X²^
Abscess during operation		36%–27.9%				21%–33.3%			.656^X²^
Perianal disease		21%–16.3%				33%–52.4%			***.003*** ^X²^
Stoma		39%–30.2%				36%–57.1%			***.038*** ^X²^
Synchronous small bowel resection		12%–9.3%				15%–23.8%			.117^X²^
Early complication		0%–0.0%				60%–95.2%			^X²^
Late complication		0%–0.0%				18%–28.6%			^X²^
Extraintestinal findings		21%–16.3%				27%–42.9%			***0.021*** ^X²^
WBC		7.2 ± 1.9	7.3	4.3–11.0		8.2 ± 1.9	8.4	5.1–12.1	***0.050*** m
Neutrophil		5.4 ± 1.5	5.3	2.5–8.9		5.4 ± 1.2	5.4	3.7–6.9	.731^m^
CRP		10.5 ± 12.6	5.4	0.4–56.0		25.6 ± 25.7	12.5	0.5–87.0	***.003*** m
ESR after 1 hour		13.4 ± 12.3	9.0	2.0–70.0		21.0 ± 21.7	11.0	4.0–97.0	.122^m^
Albumin		4.2 ± 0.5	4.3	3.1–5.2		4.0 ± 0.8	4.3	2.3–5.1	.457^m^
LDH		187.5 ± 35.3	188.0	110.0–269.0		194.0 ± 37.0	188.0	143.0–278.0	.657^m^
Time until operation (Year)		5.0 ± 4.8	3.0	0.5–19.0		5.3 ± 4.5	5.0	1.0–18.0	.544^m^
Duration of hospital stay		6.3 ± 1.5	7.0	4.0–9.0		10.4 ± 3.6	9.0	5.0–18.0	***.000*** m

ASA = American Society of Anesthesiologists, AZA = azathioprine, BMI = body mass index, CRP = C-reactive protein, ESR = erythrocyte sedimentation rate, MTX = methotrexate, TNF = tumor necrosis factor, WBC = white blood cell.

m Mann–Whitney U test/ ^X²^ Chi-square test (Fischer test).

In the univariate model, a significant (*P* < .05) effect of smoking, antibiotic use, steroid use, presence of perianal disease or extra intestinal findings, the opening of a stoma, and high CRP was observed in predicting the complication (Table 3).

## 4. Discussion

Despite all the treatments developed, approximately 2-thirds of the patients require surgical treatment. The most common reason for surgical treatment is strictures, as found in our series. Abscesses and fistulas are other common reasons for operation indication. This was also a common finding in our series since patients with treatment-resistant abscess (n = 18, 9.4%) and those with a stricture together with a perianal fistula (n = 15, 7.8%) were more likely observed. Bleeding and cancer development are less common surgical indications.^[3,5,9–11]^ Obstruction due to stenosis due to chronic recurrent inflammation and fibrosis is one of the most common indications for surgery and the most common surgical procedure is ileocecal resection. This was also consistent with our series since 153 patients (79.7%) underwent ileocecal resection only. Anastomotic margin recurrence has been reported in 34% after primary ileocecal resection.^[11]^ Endoscopic balloon dilatation should be the primary intervention for surgery in experienced centers in strictures and obstructions caused by CD; however, surgical treatment is still in the foreground in most centers.^[12,13]^ The priority of evacuation of abscesses due to CD can be left to percutaneous drainage. However, not all abscesses can be drained or accessible; therefore, the need for surgery may be inevitable.^[14]^ Although successful treatments with anti-TNF agents have been reported for CD, the standard treatment is surgery.^[10,15]^

smoking, antibiotic use, steroid use, presence of perianal disease, stoma opening during surgery, presence of extra-intestinal findings, and high CRP were significantly higher in the group with complications.

Laparoscopic surgery is more difficult in CD because of the thickened mesentery, adhesions, and potential fistula, abscess, and phlegmon. However, successful series have been reported in the literature; there are even satisfactory results in patients with a history of previous laparotomy.^[16–18]^ Despite its difficulty, laparoscopic surgery is considered standard for these patients as it is a benign disease. In the meta-analysis of Yu et al^[19]^, the method was found to be safe and applicable, although there was more blood loss, longer operation time, higher conversion rate, and longer hospitalization in laparoscopic surgery, and no significant difference was found between open surgery and laparoscopy in terms of postoperative complications. However, the low number of laparoscopic patients (n: 27, 13.8%) may have affected the reliability of the study results.

There are studies in the literature investigating the factors that may affect the development of complications after CD, similar to our study. Galata et al emphasized that low preoperative albumin levels are an important factor, and high CRP values and the emergency surgical case affect complications.^[20]^ In the study conducted by Zuo et al,^[21]^ preoperative C-reactive protein level > 10 mg/ L was determined as an independent risk factor for intraabdominal septic complications (*P* < .01); it has been stated that C-reactive protein can serve as a predictive index for intra-abdominal septic complications in Crohn patients who are candidates for surgical treatment. In another study, although obese patients had a milder severity of CD, infectious complications were found to be higher in patients with high BMI.^[22]^ In our study, in accordance with the literature, the CRP level (mean: 25.6 ± 25.7, cutoff = 8.9) in the group with complications was found to be significantly higher than the group without complications (*P* = .003).

Extraintestinal involvement findings in CD are generally observed in the joints, skin, eyes, and bile ducts. Similarly, the most common extraintestinal findings in our study were skin findings (erythema nodosum, dermatitis, and oral aphthae). It is known that the presence of extraintestinal involvement in patients with CD is associated with disease severity, disease-related mortality, and morbidity.^[23]^ Previous surgery due to inflammatory bowel disease, early diagnosis, and active smoking is associated with the development of extraintestinal symptoms.^[24]^ In our study, extraintestinal findings were found to be significantly higher in the group with complications (*P* = .021).

There are some risk factors known to increase postoperative disease recurrence in patients with CD. Active smoking, the presence of penetrating disease, recurrent operation history, and the presence of post-op residual disease are the most important of these.^[24,25]^ In our clinical series, among these risk factors, smoking and the presence of perianal and penetrating involvement were found to be higher in the group with post-op complications. Our study and many other studies in the literature reveal the necessity of motivating patients to quit smoking because of the negative effects of CD on the clinical and surgical process, as it is a modifiable risk factor.

The low number of patients in this retrospective study examining the factors affecting the development of complications after intestinal resection due to Crohn can be considered a limitation. Nevertheless, although it is the only tertiary center data and the evaluation was performed retrospectively, the prospective collection and accumulation of the data allowed many factors to be evaluated simultaneously. Another limitation to the study is the use of open approach since the laparoscopic approach became a standard due to the disease benign nature. Also, the reported literature available on this subject was scarce.

In conclusion, although medical treatment is the first choice in management, surgical methods are inevitable, especially in the treatment of complications of CD. However, after each surgery, there is a possibility of disease recurrence and surgical complications. In our study, smoking, steroid use, antibiotic use, presence of perianal disease or extraintestinal findings, and opening of a stoma appear as factors that increase the development of complications in the postoperative period. Monitoring patients with these risk factors closely during the postoperative period may help surgeons detect any possible complications earlier.

## Author contributions

**Conceptualization:** Nazim Gures, Ergin Erginoz, Yusuf Ziya Erzin.

**Data curation:** Ergin Erginoz.

**Formal analysis:** Suleyman Yildirim, Yusuf Ziya Erzin, Kagan Zengin.

**Investigation:** Ergin Erginoz.

**Methodology:** Server Sezgin Uludag.

**Project administration:** Server Sezgin Uludag, Ergin Erginoz, Suleyman Yildirim, Yusuf Ziya Erzin.

**Resources:** Server Sezgin Uludag.

**Supervision:** Nazim Gures, Server Sezgin Uludag, Kagan Zengin.

**Validation:** Suleyman Yildirim, Kagan Zengin.

**Visualization:** Nazim Gures, Suleyman Yildirim.

**Writing – original draft:** Ergin Erginoz.

**Writing – review & editing:** Nazim Gures, Yusuf Ziya Erzin, Kagan Zengin.
